# Supplementary material for: Availability, acceptability and uptake of Sexual Reproductive Health interventions for young people with disabilities in Sub Saharan Africa: A scoping review protocol
Source: PLoS One. 2025 May 21;20(5):e0324364. doi: 10.1371/journal.pone.0324364 (PMC12094729; doi:10.1371/journal.pone.0324364)
Supplement: Attachment — Submitted filename: Review of the Article.docx [file pone.0324364.s002.docx]

**Review of the Article**

PONE-D-25-02097
Availability, acceptability and uptake of Sexual Reproductive Health interventions for young people with disabilities in Sub Saharan African: A scoping review protocol
PLOS ONE

I want to congratulate the authors for selecting an interesting topic. The manuscript is well-written. However, I would like to provide a few suggestions for revising the manuscript.

Title and Abstract

The title accurately reflects the focus of the study: “Availability, acceptability, and uptake of sexual reproductive health interventions for young people with disabilities in Sub-Saharan Africa.” However, there is a minor grammatical issue in the title: "African" should be "Africa" (i.e., *Sub-Saharan Africa* instead of *Sub-Saharan African*).

The abstract clearly states the research problem, methodology, and expected contribution of the study. However, the research gap could be articulated more strongly. For instance, instead of broadly stating that knowledge is limited, the authors should specify which aspects of SRH interventions remain particularly underexplored (e.g., policy gaps, service delivery models).

The discussion in the abstract could better explain the practical implications of the study, particularly how it informs policy and programming.

Introduction

The introduction effectively frames the importance of SRH services for young people with disabilities. It provides statistical data and international policy references (e.g., CRPD, ICESCR), making it a strong foundation. However, some areas need improvement:

i. Several points (e.g., stigma, inaccessibility of SRH services, exclusion from interventions) are mentioned multiple times. A more concise presentation of these barriers would improve readability.
ii. Concepts like "buy-in," "existential realities," and "coterie of challenges" could be simplified or replaced with more precise language to enhance accessibility.
iii. The introduction focuses on challenges or barriers to accessing SRH interventions (stated broadly) without addressing availability as an important variable. The study fails to highlight the existing interventions for the general population and those specific to young people with disabilities. Discussing availability first would provide a clearer framework for assessing accessibility and mapping out which interventions work and which do not. The scoping review would be strengthened by incorporating this important variable.

Methodology

The methodology is robust and follows the Arksey and O’Malley scoping review framework. However, a few areas need clarification:

While a list of databases is provided, the authors should include details on:

i. How search terms were combined using Boolean operators (e.g., AND, OR).
ii. Whether any language restrictions were applied beyond English.
iii. Whether gray literature sources (e.g., NGO reports, government documents) will be systematically appraised.

The authors state that two reviewers will independently screen articles, but how will discrepancies be handled if the third reviewer is unavailable?

Will any software (e.g., Rayyan, Covidence) be used to ensure consistency in the review process?

The authors mention CASP checklists and RoB tools, but scoping reviews typically do not conduct quality appraisal. They should clarify why these tools are relevant in this context.

The manuscript states that no ethical approval is required because only secondary data is used. However, it would be beneficial to acknowledge any potential ethical concerns regarding the representation of people with disabilities in research.

Discussion

The discussion explains the gaps in SRH services for young people with disabilities and the potential contribution of the review. However:

The statement: "Our study will fill knowledge gaps on availability, acceptability, and uptake of SRH interventions for young people with disabilities." is overly broad. This gap has not been clearly established based on the review, and it needs to be explicitly defined. The authors should specify which aspects of these interventions they aim to address, as previously mentioned. Importantly, a scoping review does not evaluate effectiveness; it maps available literature.

The exclusion of non-English studies is noted as a limitation, but the authors should discuss how this might bias the findings (e.g., missing key studies from Francophone and Lusophone African countries). This exclusion could lead to an incomplete review.

"There is paucity of knowledge" should be corrected to "There is a paucity of knowledge."

Some long sentences could be broken down for better clarity. The paper would benefit from an editorial review for grammatical errors and sentence structure improvements.

Some claims, especially regarding barriers to SRH services, stigma, and discrimination, lack proper citations. Every statement making a claim about accessibility or service delivery should be backed by references.

The manuscript does not explicitly outline areas for further research. A strong scoping review should conclude with recommendations on which SRH interventions need further study, the need for primary research in specific areas where literature is lacking, and how policies and programmes should be tailored based on the findings.

The manuscript is well-structured and covers an important topic. However, addressing the issues of availability, clarifying methodological details, tightening the discussion, and ensuring proper citation of claims will significantly improve the quality of the paper. Additionally, a final editorial review for grammar and clarity is recommended.
