# Supplementary material for: Availability, acceptability and uptake of Sexual Reproductive Health interventions for young people with disabilities in Sub Saharan Africa: A scoping review protocol
Source: PLoS One. 2025 May 21;20(5):e0324364. doi: 10.1371/journal.pone.0324364 (PMC12094729; doi:10.1371/journal.pone.0324364)
Supplement: Attachment — Submitted filename: Response to comments.docx [file pone.0324364.s004.docx]

| QUERY | RESPONSE |
| --- | --- |
| ACADEMIC EDITOR AND JOURNAL REQUIREMENTS | |
| 1. Please ensure that your manuscript meets PLOS ONE's style requirements, including those for file naming. | We have formatted our manuscript to meet PLOS ONE’s style requirements including those for file naming. |
| 2. In your cover letter, please confirm that the research you have described in your manuscript, including participant recruitment, data collection, modification, or processing, has not started and will not start until after your paper has been accepted to the journal (assuming data need to be collected, or participants recruited specifically for your study). In order to proceed with your submission, you must provide confirmation. | We have confirmed in the cover letter that participant recruitment, data collection and processing has not started and will only commence after the publication of the protocol |
| 3. Please review your reference list to ensure that it is complete and correct. If you have cited papers that have been retracted, please include the rationale for doing so in the manuscript text or remove these references and replace them with relevant current references. Any changes to the reference list should be mentioned in the rebuttal letter that accompanies your revised manuscript. If you need to cite a retracted article, indicate the article’s retracted status in the References list and include a citation and full reference for the retraction notice. | We have reviewed our references list, and we confirm that it is correct and complete, and we have added new references as suggested by reviewer 2 “Some claims, especially regarding barriers to SRH services, stigma, and discrimination, lack proper citations. Every statement making a claim about accessibility or service delivery should be backed by references**”**  **Added references**  1. World Health Organisation. Disability 2025 [Available from: <https://www.who.int/health-topics/disability#tab=tab_1>.  2. Kassa TA, Luck T, Bekele A, Riedel-Heller SG. Sexual and reproductive health of young people with disability in Ethiopia: a study on knowledge, attitude and practice: a cross-sectional study. Globalization and health. 2016;12(1):5.  3. Vivet N, de La Rochebrochard E, Martin P. Young people with disabilities and their sexual health: a descriptive review of needs, recommendations and interventions. BMC public health. 2025;25(1):930.  4. Meherali S, Rehmani M, Ali S, Lassi ZS. Interventions and Strategies to Improve Sexual and Reproductive Health Outcomes among Adolescents Living in Low- and Middle-Income Countries: A Systematic Review and Meta-Analysis. Adolescents. 2021;1(3):363-90.  5. Chipako I, Singhal S, Hollingsworth B. Impact of sexual and reproductive health interventions among young people in sub-Saharan Africa: a scoping review. Front Glob Womens Health. 2024;5:1344135.  6. Desrosiers A, Betancourt T, Kergoat Y, Servilli C, Say L, Kobeissi L. A systematic review of sexual and reproductive health interventions for young people in humanitarian and lower-and-middle-income country settings. BMC public health. 2020;20(1):666.  7. Sanyang Y, Sanyang S, Ladur AN, Cham M, Desmond N, Mgawadere F. Are facility service delivery models meeting the sexual and reproductive health needs of adolescents in Sub-Saharan Africa? A qualitative evidence synthesis. BMC health services research. 2025;25(1):193.  8. Wado YD, Bangha M, Kabiru CW, Feyissa GT. Nature of, and responses to key sexual and reproductive health challenges for adolescents in urban slums in sub-Saharan Africa: a scoping review. Reprod Health. 2020;17(1):149.  9. Obiezu-Umeh C, Nwaozuru U, Mason S, Gbaja-Biamila T, Oladele D, Ezechi O, et al. Implementation Strategies to Enhance Youth-Friendly Sexual and Reproductive Health Services in Sub-Saharan Africa: A Systematic Review. Front Reprod Health. 2021;3:684081.  10. Rugoho T, Ganle J, Stein M, Groce N, Wright P, Broerse JWE. NGOs and the Promotion of the Sexual and Reproductive Rights of Girls and Young Women with Disabilities in Zimbabwe. Social Policy and Society. 2023;2023:1-13.  11. UNFPA. Situational Analysis: Access to Sexual and Reproductive Health and Rights (SRHR) for Young Persons with Disabilities in East and Southern Africa 2017. |
| REVIEWER 1 | |
| 4. Title and Abstract   The title accurately reflects the focus of the study: “Availability, acceptability, and uptake of sexual reproductive health interventions for young people with disabilities in Sub-Saharan Africa.” However, there is a minor grammatical issue in the title: "African" should be "Africa" (i.e., *Sub-Saharan Africa* instead of *Sub-Saharan African*). | Thank you for the comment, we have corrected to Sub-Saharan Africa. |
| 5. Title and Abstract   The abstract clearly states the research problem, methodology, and expected contribution of the study. However, the research gap could be articulated more strongly. For instance, instead of broadly stating that knowledge is limited, the authors should specify which aspects of SRH interventions remain particularly underexplored (e.g., policy gaps, service delivery models). | Our scoping review is targeting service delivery interventions for young people with disabilities. We have specified in the abstract that there is a paucity of knowledge on availability, acceptability and uptake of SRH service delivery interventions for young people with disabilities. |
| 6. Title and Abstract   The discussion in the abstract could better explain the practical implications of the study, particularly how it informs policy and programming. | For the SRH service delivery interventions available, our scoping review will demonstrate which service delivery interventions work or do not work (according to mapped studies) as well as gaps in SRH service delivery interventions for young people with disabilities. This information is useful for policy making and for designing effective SRH service delivery interventions targeting young people with disabilities.  The scoping review aims to map the availability of sexual and reproductive health (SRH) service delivery interventions for young people with disabilities. This mapping of evidence has the potential to identify whether there is a need for specific SRH service delivery interventions tailored to this group. |
| 7. Introduction   Several points (e.g., stigma, inaccessibility of SRH services, exclusion from interventions) are mentioned multiple times. A more concise presentation of these barriers would improve readability. | As rightly noted, these barriers were spread in several paragraphs. We have since revised and put all barriers in paragraph 4. We believe this should be able to improve readability. |
| 8. Introduction   Concepts like "buy-in," "existential realities," and "coterie of challenges" could be simplified or replaced with more precise language to enhance accessibility | We have simplified “buy in” to “support”, “existential realities” to “unique experiences” and “coterie of challenges” to “several challenges” |
| 9. Introduction   The introduction focuses on challenges or barriers to accessing SRH interventions (stated broadly) without addressing availability as an important variable. The study fails to highlight the existing interventions for the general population and those specific to young people with disabilities. Discussing availability first would provide a clearer framework for assessing accessibility and mapping out which interventions work and which do not. The scoping review would be strengthened by incorporating this important variable. | In line 99-113 (tracked version), we have discussed the implementation of SRH service delivery interventions for young people as a category as well delivery of SRH services for YPWD. We have specified that NGOs tend to be on the forefront of providing SRH services for YPWD. |
| 10. Methodology   While a list of databases is provided, the authors should include details on:   i. How search terms were combined using Boolean operators (e.g., AND, OR). ii. Whether any language restrictions were applied beyond English. iii. Whether gray literature sources (e.g., NGO reports, government documents) will be systematically appraised. | i. In the stage 2, we have clarified that the search terms will be combined with operator ‘OR’ and concepts by operator ‘AND’. ii. We have clarified the exclusion of non-English studies. In our team, we do not have competency in other languages. We also acknowledge that, while tools like Google Translate can be used for non-English studies, we worry some concepts may not always translate well which may affect the quality of the review, hence our focus on studies written in English. iii. We have highlighted in stage 2 that we are going to search Open Grey database for reports, doctoral dissertations, official publications and other types of grey literature. |
| 11. Methodology   The authors state that two reviewers will independently screen articles, but how will discrepancies be handled if the third reviewer is unavailable? | In stage 2 of study selection, we have clarified that disagreements between the two reviewers will be resolved through reviewers’ discussion or arbitration by a third person (ON). Please see line 213-214 of tracked version. |
| 12. Methodology   Will any software (e.g., Rayyan, Covidence) be used to ensure consistency in the review process? | We have mentioned in Stage 2 that we will use Covidence to ensure consistency in the review process. Please see line 211, tracked version. |
| 13. Methodology   The authors mention CASP checklists and RoB tools, but scoping reviews typically do not conduct quality appraisal. They should clarify why these tools are relevant in this context. | Since scoping reviews do not typical conduct quality appraisal, we have decided to drop the quality appraisal for this study. |
| 14. Methodology   The manuscript states that no ethical approval is required because only secondary data is used. However, it would be beneficial to acknowledge any potential ethical concerns regarding the representation of people with disabilities in research. | Thank you for raising this pertinent issue. Throughout the scoping review we have used language that is not demeaning to young people living with disabilities, we have referred them as ‘young people with disabilities’ and avoided demeaning terms like crippled etc. |
| 15. Discussion   The statement: "Our study will fill knowledge gaps on availability, acceptability, and uptake of SRH interventions for young people with disabilities." is overly broad. This gap has not been clearly established based on the review, and it needs to be explicitly defined. The authors should specify which aspects of these interventions they aim to address, as previously mentioned. Importantly, a scoping review does not evaluate effectiveness; it maps available literature. | We have revised the discussion section to capture that currently it is unknown which SRH service delivery interventions are available for young people. Mapping evidence will determine whether SRH interventions for young people with disabilities are needed. Mapping evidence on which SRH service delivery interventions work and do not work can inform the designing of effective service delivery interventions for young people. |
| 16. Discussion   The exclusion of non-English studies is noted as a limitation, but the authors should discuss how this might bias the findings (e.g., missing key studies from Francophone and Lusophone African countries). This exclusion could lead to an incomplete review. | We have strengthened the limitation and specified that the exclusion of non-English papers will introduce bias in findings. |
| 17. Discussion   "There is paucity of knowledge" should be corrected to "There is a paucity of knowledge." | We have corrected the phrase to read “there is a paucity of knowledge” |
| 18. Discussion   Some long sentences could be broken down for better clarity. The paper would benefit from an editorial review for grammatical errors and sentence structure improvements. | We have enlisted the services of a language editor to revise the paper and improve sentence structure and readability. |
| 19. Discussion   Some claims, especially regarding barriers to SRH services, stigma, and discrimination, lack proper citations. Every statement making a claim about accessibility or service delivery should be backed by references. | We have added citations to statements making claims about accessibility and service delivery. Please see lines (tracked version) 87, 89, 102, 107, 108, 128, 134, 136, 141, 247. |
| 20. Discussion   The manuscript does not explicitly outline areas for further research. A strong scoping review should conclude with recommendations on which SRH interventions need further study, the need for primary research in specific areas where literature is lacking, and how policies and programmes should be tailored based on the findings. | Thank you for the comment, we have made changes in the discussion section (line 258-266) emphasising that based on the consolidated evidence from the scoping review we will be able to make recommendations whether there is any need for specific SRH interventions for YPWD as well any areas needing primary research. |
| 21. Discussion   The manuscript is well-structured and covers an important topic. However, addressing the issues of availability, clarifying methodological details, tightening the discussion, and ensuring proper citation of claims will significantly improve the quality of the paper. Additionally, a final editorial review for grammar and clarity is recommended | Thank you for the comment, we have addressed the concerns raised as we were addressing the queries flagged by the reviewer. |
| REVIEWER 2 | |
| 22. Introduction   Provide a date/year for this data: “The World Health Organisation (WHO) estimates that there are 1 billion people living with disabilities globally, including 220 million young people.” | A year (2025) has been added to this statement. |
| 23. Introduction   Paragraph 2 is too long. A paragraph may not exclude 12 lines. | We have shortened the paragraph by splitting it into 2. |
| 24. Introduction   This statement appears unclear: “SRH interventions for young people with disabilities fail because of lack of buy-in as well as being divorced from their contextual experiences and realities of living with disabilities.” | We have tried to simplify the statement to read “SRH interventions for young people with disabilities often fail because they lack support and do not consider the unique experiences and challenges young people with disabilities face in their daily lives” |
| 25. Methods   The disability context should include the “people living with disability” keyword. | Added on the context section, table 1 |
| 26. Methods   You may have to include MeSH terms for the constructs. For example, disability has the "Persons with Disabilities"[Mesh] MeSH term, while sub-Saharan Africa has a MeSH in PubMed: "Africa South of the Sahara"[Mesh]. | We have included MeSH terms for various constructs of the study. |
| 27. Methods   There is a need to incorporate * to explode some terms. For example, some authors may use the phrase “sexual behaviour” (British language), while others may use “sexual behavior” (American English). To capture both simultaneously, you may include the * and write it as “sexual behavi*”. Another example is youth. I would write youth* to capture both youth and youths. | The recommendation to use* to explode some terms has been applied accordingly. |
| 28. Methods   The number of countries in the sub-saharan Africa concept is incomplete. Eswatini, Mauritania, and Mauritius are missing. Furthermore, please include “West Africa,” “East Africa,” “Southern Africa,” OR “Central Africa” because some authors focus on multiple countries within the same region and report regions rather than individual countries. | Eswatini, Mauritania, and Mauritius as well as West Africa, East Africa, Southern Africa and Central Africa were added. |
| 29. Methods   Single terms such as disabled, Congo, etc. do not need to be in quote. “ “ is used for two or more words to make them “one” or turn them into phrases. | We have removed “” from single terms and used “” for two or more words to make them one or turn them into phrases. |
| 30. Methods   Please note that Embase uses emtree (similar to MeSH) terms. Please use the available emtree in Embase for a more comprehensive search. | Thank you, we have used the available emtree in Embase for a more comprehensive search. |
| 31. Methods   I can see from Table 2 that the authors plan to remove non-English articles. I strongly discourage that because there are tools that can translate documents now. Google Translate does a good job, too. So, this is no longer a valid exclusion criterion because non-English articles matter. | Thanks for the suggestion to use Google Translate, however we worry that it may not always accurately convey the nuances of scientific terminology or complex ideas, potentially leading to misinterpretations. Some concepts may not always translate well which may affect the quality of the review. |
| 32. Methods   Is there a justification for limiting the search to 2000-2025? | We believed the period allows us to map contemporary evidence which is key to inform policy and practice. |
| 33. Discussion   I discussed the issue of excluding non-English studies. | Thanks for the suggestion to use Google Translate, however we worry that it may not always accurately convey the nuances of scientific terminology or complex ideas, potentially leading to misinterpretations. Some concepts may not always translate well which may affect the quality of the review. |
| REVIEWER 3 | |
| 34. I appreciate the invitation to perform this review. In my opinion, the protocol was satisfactorily written with all the necessary components needed included and detailed information of step-by-step procedures of the methodology included. it is a well written protocol explicit and detailed | Thank you for the affirming comments. We have additionally strengthened the protocol following guidance from reviewer 1 and reviewer 2. |
